# A comparison of snack serving sizes to USDA guidelines in healthy weight and overweight minority preschool children enrolled in Head Start

**DOI:** 10.1186/s40608-016-0116-2

**Published:** 2016-08-27

**Authors:** Andrea Charvet, Kathryn Brogan Hartlieb, Yulyu Yeh, K.-L. Catherine Jen

**Affiliations:** 1 Department of Dietetics and Nutrition, Robert Stempel College of Public Health and Social Work, Florida International University, 11200 SW 8th Street, Miami, FL 33199 USA; 2 Department of Nutrition and Food Science, College of Liberal Arts and Sciences, Wayne State University, 3009 Science Hall, Detroit, MI 48202 USA

**Keywords:** Snack, Serving size, USDA, Overweight, Children, Minority, Head Start

## Abstract

**Background:**

Obesity disproportionately affects children from low-income families and those from racial and ethnic minorities. The relationship between snacking and weight status remains unclear, although snacking is known to be an important eating episode for energy and nutrient intake particularly in young children. The purpose of this pilot study was to examine the snack intake of minority preschool children enrolled in the Head Start Program in four centers in Detroit, Michigan, and investigate differences by child weight status.

**Methods:**

This secondary data analysis used snack time food observation and anthropometric data from a convenience sample of 55 African American children (44 % girls, mean age = 3.8 years). Snack intake data was obtained over a mean of 5 days through direct observation of children by dietetic interns, and later converted into food group servings according to the United States Department of Agriculture (USDA) meal patterns and averaged for each child. Height and weight measurements were systematically collected and BMI-for-age percentiles were used to classify children into weight categories. One sample, paired samples and independent samples *t*-tests were performed to test for differences within and between means.

**Results:**

Based on BMI-for-age percentiles, 72.7 % of the sample was under/healthy weight and 27.3 % was overweight/obese. Average (mean ± SD) intake of milk (0.76 ± 0.34) and overall fruits/vegetables (0.77 ± 0.34) was significantly lower than one USDA serving, while average intake of grains and breads (2.04 ± 0.89), meat/meat alternates (2.20 ± 1.89) and other foods (1.43 ± 1.08) was significantly higher than one USDA serving (*p* ≤ 0.05). Children ate more when offered canned versus fresh fruits (0.93 ± 0.57 vs. 0.65 ± 0.37, *p* = 0.007). Except for a significantly higher milk intake in the overweight/obese group compared to the under/healthy weight group (0.86 ± 0.48 vs. 0.72 ± 0.27, *p* = 0.021], no relationship was found between snack food intake and weight category. Only in the overweight/obese group was the intake of milk and fresh fruits not significantly different than one USDA serving.

**Conclusions:**

Findings suggest that regardless of weight status low-income minority preschool children are consuming larger serving sizes when offered less healthy versus healthier snack foods. Continued efforts should be made to provide healthful snack foods at preschool settings to prevent obesity and promote healthier food habits.

## Background

The incidence of childhood obesity increased dramatically in the past 30 years, and has become a major concern in children’s health outcomes. Data from the 2011–2012 National Health and Nutrition Examination Survey (NHANES) show that 22.8 % of all children ages 2 to 5 years old are overweight or obese [1]. Despite a leveling off or slight decrease in childhood obesity rates in some populations [1–3], the obesity rates remain high particularly among children from low-income families and those from racial and ethnic minorities. Among African American children aged 2–5 years, despite a decrease in combined overweight and obesity rates between 2007–2008 and 2011–2012, obesity alone rates remained unchanged at approximately 11.4 % [1, 4].

Childhood obesity has serious psychosocial and physical consequences. Overweight and obese children are more likely to become targets of discrimination, which can lead to depression [5, 6]. Overweight or obese children have a significantly higher risk of becoming overweight or obese adults when compared to healthy weight children [7–9]. Obesity is a risk factor for many chronic diseases, including diabetes, coronary heart disease, dyslipidemias, asthma, certain cancers, and arthritis [10–12]. Preschool children from minority families are at a higher risk for elevated cardiovascular disease biomarkers [13]. For example, Brogan et al. reported that at least 40 % of 3–5 year-old children in a sample composed of 92.5 % African Americans presented borderline or high-risk levels of high-density lipoprotein and triglycerides [14]. Obesity in childhood also generates a large economic burden. Children who are diagnosed with obesity have significantly higher healthcare costs when compared to healthy weight children [15]. Furthermore, if obesity and overweight prevalence in the U.S. population continues to increase at current rates, the total healthcare costs are estimated to more than double during each subsequent decade [16]. The high incidence and serious consequence of childhood obesity at an early age, especially among minorities, highlights the importance of strategies targeting a healthy weight on minority groups in the preschool years.

While the causes of obesity include environmental and individual factors [2], this paper will focus on snacking, which is likely to play an important role in the development of overweight and obesity [17, 18]. Snacks can serve as a potential way to introduce new foods and to offer nutrients not consumed at other meals. Snacking has been defined in different ways [19]. Given the range of definitions, for the purpose of this study a snack was defined as a meal served between lunch and dismissal from childcare at Head Start centers.

Healthy snacking between the three main meals has been suggested as a way to improve the quality of the food intake in children even though the relationship between snacking behaviors and weight status in children remains unclear [20, 21]. A literature review by Larson and Story addressing the implications of snacking on weight status found mixed results, the majority of cross-sectional and longitudinal studies either found no relationship between snacking behavior and weight status or found that children consuming snacks between meals were less likely to be obese [21]. Maffeis and colleagues assessed snack intake in 8- to 10-year old children and its relationship to body size and reported that obese and overweight children are not eating significantly more snacks than normal weight children, but are eating significantly more energy dense snacks, with a preference to those with a salty taste [20].

Findings from a study by Evans et al. suggest that the number of snacks is positively associated with a better diet quality as evidenced by an increased Healthy Eating Index (HEI) 2005 score in elementary school-age children [22]. Snacking frequency and the contribution of energy from snacks to total daily energy intake has been increasing over the years. Cross et al. evaluated snacking patterns among adults and children in the U.S. and reported that 92.6 % of elementary school aged children snacked once a day or more [23]. Between the years of 1977 and 1996 the percent of preschool aged children snacking at least once every day increased from 79 to 94 %, and the mean number of snacks per day increased significantly from 1.73 to 2.29, with the proportion of energy from snacks increasing from 19 to 24 % [24]. New data from the United States Department of Agriculture (USDA) reports that in 2011–2012 snacks contributed to approximately 30 % of total daily energy intake in preschool aged children [25]. Given the high prevalence of snacking among young children and its potential impact on diet quality, snacking behaviors continue to be an important area of study.

Children ages six and younger spend, on average, 25 h per week in child-care settings, with approximately two out of three preschool-aged children being enrolled in some type of non-parental childcare program [26]. It has been suggested that childcare experiences may have a significant influence on eating habits, and consequently on weight status in childhood [27]. Head Start is a Federal program that provides preschool services in center-based settings for low-income children between the ages of 0 and 5 years [28], therefore it was a good setting for examining snacking in low-income minority children.

Understanding differences in snacking based on weight status may provide valuable data for obesity prevention interventions. The purpose of this pilot study was two-fold, first to examine snack intake of minority preschool children enrolled in the Head Start Program in four centers in Detroit, Michigan, in relationship to USDA serving size guidelines for reimbursable snacks; and second, to investigate differences in snacking by child weight status. It was hypothesized that snack intake would be significantly different between children who are underweight and healthy weight and children who are overweight and obese.

## Methods

### Setting and participants

This secondary data analysis used de-identified snack time food observation and anthropometric data collected at baseline from the intervention study “Healthy Kids Healthy Lives” [29], during the fall of 2008. The Wayne State University Institutional Review Board approved the original study protocol and written informed consent was obtained from all caregivers. The study was conducted through a Head Start system with 15 sites in the city of Detroit, Michigan.

A convenience sample of 60 African American children aged 3 to 5 years were recruited from four of the Head Start sites. To be included in the study children were eligible to participate in the Head Start program, which include meeting federal poverty guidelines to evaluate family income, as well as consenting to participate and having attended preschool on food observation days. Children with special needs were excluded from study enrollment as this may impact their snack intake and body weight. The Institutional Review Board at Florida International University approved the current study.

### Snack intake

Snack intake was obtained from the children through direct observation by dietetic interns (DI) twice per week during their community nutrition internship rotation, between October 14th and December 12th, 2008. Attendance of children to the Head Start center is variable. Snack intake observation was recorded for all children present the days the DI’s were at the centers.

Meals served at the Head Start centers are family-style, meaning children select their own portions and serve themselves [30]. Prior to completing the food observations, the DIs reviewed visual portion size materials. Groups of 3 to 8 children sat in semicircular tables with a Head Start teacher leading the family-style snack period. The DIs sat in an unobtrusive location during the snacking episodes at the Head Start sites and recorded on paper the amount of each snack food the child consumed. Portions consumed were determined by visual estimates of volume since weighting foods was not feasible at the sites.

Head Start centers provide meals and snacks free of charge. In order for the centers to receive reimbursement from the U.S. government, they are required to follow certain guidelines established by the USDA for the meals they serve. These guidelines require that meals and snacks include a minimum number of age-appropriate servings from 4 food groups: milk, fruits and vegetables, grains and breads, and meat and meat alternates. A reimbursable snack must include at least two of the four groups [31]. Table 1 provides a summary of the food groups and serving sizes per USDA regulations for children ages 3 through 5 years, along with examples of snack foods served across the four Head Start centers observed for this study. Because the snack foods served at the four observed centers were similar, they are grouped together when reported.

**Table 1:** USDA reimbursable snack pattern for child care^a^, ages 3–5, with examples of snacks served

Food group	Minimum serving size	Examples of snacks served at the Head Start centers
Milk		
Fluid milk	1/2 cup^b^	2 % reduced fat milk
Vegetables/Fruits		
Full strength juice or	1/2 cup	Orange slices, grapes, applesauce, canned pineapple, fruit cocktail, banana, canned peaches, carrots
Fruit and/or vegetable	1/2 cup	
Grains/Bread^c^		
Bread or	1/2 slice	Pumpkin bread, whole-wheat bagel, raisin bran muffin, blueberry muffin, oatmeal cookie, peanut butter cookie, whole-wheat crackers, animal crackers, Rice Krispies, Honey Nut Cheerios, Raisin Bran, cupcake, yellow cake, tortilla chips
Cornbread, biscuits, rolls, muffins or	1/2 serving	
Cold dry cereal or	1/4 cup	
Hot cooked cereal or	1/4 cup	
Cooked pasta or noodles or grains	1/4 cup	
Meat/Meat Alternate		
Meat or poultry or fish^d^ or	1/2 oz	Sliced turkey, turkey and beans chili, cheddar cheese, cottage cheese, scrambled eggs, peanut butter, Light & Fit yogurt, vanilla yogurt, strawberry yogurt
Alternate protein product or	1/2 oz	
Cheese or	1/2 oz	
Egg^e^ or	1/2 egg	
Cooked dry beans or peas or	1/8 cup	
Peanut or other nut or seed butter or	1 Tbsp.	
Nuts and/or seeds or	1/2 oz	
Yogurt^f^	2 oz or 1/4 cup	

^a^Adapted from the U.S. Department of Agriculture (USDA), Food and Nutrition Service (FNS). Federal regulations, part 226-Child and Adult Care Food Program [33]

^b^A cup means a standard measuring cup

^c^Breads and grains must be made from whole-grain or enriched meal or flour. Cereal must be whole-grain or enriched or fortified

^d^A serving consists of the edible portion of cooked lean meat or poultry or fish

^e^One-half egg meets the required minimum amount (1 oz or less) of meat alternate

^f^Yogurt may be plain or flavored, unsweetened or sweetened

Certain food items do not meet the requirement for any of the four food groups, but may still be used. The USDA Food Buying Guide for Child Nutrition Programs uses the term “other foods” and includes such items since they are frequently used as condiments and seasonings, to round out the meal, to improve acceptability, and to satisfy children’s appetites [32]. Other foods served at the Head Start sites included items such as cream cheese, cheese dip, and ice cream.

### Anthropometric measurements

Weight and height measurements were collected from all participating children before beginning the study. Trained graduate student data collectors weighed children without coats and shoes using a portable digital scale (Tanita Model BC551). Two measures were obtained from each child. If the two readings were within 0.1 kg, an average was taken and used as the body weight. If the two readings differed by more than 0.1 kg, a third reading was taken and readings were averaged. Heights were obtained using a portable stadiometer (Seca 214, Seca North America East, Hanover, MD). Two measurements without shoes were recorded. If the two readings were within 0.2 cm of each other the readings were averaged and recorded. If the difference was more than 0.2 cm, a third measurement was taken before averaging. Weight and height measurements were used to calculate body mass index (BMI). BMI-for-age percentiles were used to categorize the child as underweight (<5 %), healthy weight (5–84.9 %), overweight (85–94.9 %) and obese (≥95 %), following the Centers for Disease Control and Prevention (CDC) guidelines for BMI-for-age weight status categories [33]. Details on the method of calculating BMI and its expression as BMI-for-age percentiles can be found on the CDC website [33].

### Data analysis

For the purpose of this study, snack intake was converted into food group servings based on the USDA meal patterns as the minimum amount of food that can be served to comply with the licensing standards for adequate nutrition for children between 3 and 5 years of age [34]. To assist in determining serving sizes for foods not included on the USDA meal patterns the authors contacted the U.S. Department of Agriculture Food and Nutrition Services and the Bureau of Child Care Food Program. Per recommendations the USDA Food Buying Guide for Child Nutrition Programs was used [32]. Food groups included milk, fruits and vegetables, grains and breads, meat and meat alternates, and other foods.

In order to explore snack food intake quality, food groups were further categorized by their healthfulness. The fruits and vegetables food group was subdivided into fresh fruits and canned fruits as indicated by the measurement. In the instances that fresh vs. canned fruit was not specified by the DI (66 % of fruit intake records) pineapple, pears, peaches and apricots were assigned canned fruit, while bananas, apples, grapes and oranges were assigned fresh fruit. The grains and bread food group was subdivided into low sugar/low fat grains and breads and high sugar/high fat grains and breads. Low sugar/low fat grains and breads included breakfast cereal, wheat crackers, wheat bagels, and whole wheat breads. High sugar/high fat grains and breads included cookies, graham and animal crackers, cupcakes and cakes. Though this study did not measure nutrient quality, it can be assumed that fresh fruits and low sugar/low fat grains and breads tend to be healthier than canned fruits and high sugar/high fat grains and breads.

Average snack intake for each food component was calculated per individual. For each child, an absent day was not included in the data analysis. Meanwhile, a day the child was present at the Head Start center but did not consume a certain food component that was offered, intake was computed as “zero” and that day was counted for data analysis.

BMI categories were grouped into dichotomized variables to allow for comparisons by weight status. Children who were underweight and healthy weight were grouped into a variable labeled underweight/healthy weight and children who were overweight and obese were grouped into a variable labeled overweight/obese.

Data was entered into the computer and analyzed using the Statistical Package for Social Sciences version 21 software (SPSS, IBM Corp, Armonk, NY). A significance level of 5 % was used on all statistical tests performed. One sample *t*-tests were conducted to test for differences comparing to USDA serving sizes. Paired samples *t*-tests were conducted to test for differences within fresh fruits and canned fruits, and low sugar/low fat grains and breads and high sugar/high fat grains and breads. To test for equality of means for intake of each food component by weight category, independent samples *t*-tests were performed.

## Results

### Demographics

From the 60 children recruited, a total of 55 African American children enrolled in the Head Start program were included in the study. Two children enrolled were excluded from the study due to missing anthropometric data, and three enrolled children were excluded due to missing snack intake data for the days they were present at the Head Start center. Study participants had a mean age of 46 months (3.8 years) ± 7.95, with 31 boys (56 %) and 24 girls (44 %). According to the BMI-for-age percentiles five children were underweight (9.1 %), 35 were at healthy weight (63.6 %), seven were overweight (12.7 %), and eight were obese (14.5 %). When weight categories were grouped, 72.7 % (*n* = 40) children were underweight/healthy weight, and 27.3 % (*n* = 15) were overweight/obese.

### Snack quantity and quality

Snack time observation data recorded for the days each child was present at the Head Start site varied from 1 to 18 days of data record, because of children’s variability in attendance. On average a child was present for 5 days (median = 4 days, SD = 3.90). Milk (2 %) was the only food group offered daily. Fruits or vegetables were offered 13 out of 18 days (fresh fruits 5 days, canned fruits 8 days, vegetables 1 day, offered together with a fruit), grains and breads offered 11 days (low sugar/low fat 6 days, high sugar/high fat 5 days), meat and meat alternates 5 days and other foods were offered three out of the 18 days.

Table 2 reports the children’s mean snack intake of each food group compared to one USDA serving. The mean snack intake of the milk and the fruits and vegetables group was significantly lower than one USDA serving (*p* < 0.001). However, the mean snack intake of grains and breads (*p* < 0.001), meats and meat alternates (*p* = 0.001), and other foods (*p* = 0.047) was significantly higher than one USDA serving. When the canned and fresh fruit categories were examined separately, only the mean snack intake of fresh fruits was significantly lower than one USDA serving (*p* < 0.001). Canned fruits mean snack intake was not significantly different from one USDA serving (*p* = 0.124). Mean snack intake of low sugar/low fat and high sugar/high fat grains and breads were both greater than one USDA serving.

**Table 2:** Intake (mean ± SD) per snacking episode in number of servings, compared to one USDA serving

Food group	n^a^	Mean intake ± SD	*p*-value
Milk	55	0.76 ± 0.34	<0.000**
Fruits and Vegetables Overall	53	0.77 ± 0.34	<0.000**
Fresh fruits	40	0.67 ± 0.34	<0.000**
Canned fruits	43	0.87 ± 0.53	0.124
Grains and Breads Overall	51	2.04 ± 0.89	<0.000**
Low sugar/low fat^b^	44	1.75 ± 0.71	<0.000**
High sugar/high fat^c^	42	2.12 ± 1.10	<0.000**
Meat and Meat Alternates^d^	34	2.20 ± 1.89	0.001**
Other Foods^e^	27	1.43 ± 1.08	0.047*

Statistics are based on all observations with valid data

^a^n represents the total number of children analyzed per food group. Milk was the only food group offered daily, for which all participating children had a record of intake

^b^Low sugar/low fat grains and breads included breakfast cereal, wheat crackers, wheat bagels, whole wheat breads

^c^High sugar/high fat grains and breads included cookies, graham and animal crackers, cupcakes and cakes

^d^Meat and meat alternates included cheeses, yogurt, eggs, deli meats, and peanut butter

^e^Other foods included cream cheese, cheese dip, and ice cream

* 95 % significance level

** 95 % significance level

When comparing child snack intake by healthy and less healthy categories, paired samples test showed that children had significantly higher fruit intake when offered canned fruits vs. fresh fruits (0.93 ± 0.57 vs. 0.65 ± 0.37 servings respectively, *p* = 0.007). No difference was found when comparing mean snack intake of low sugar/low fat grains and breads with high sugar/high fat grains and breads (1.72 ± 0.71 vs. 1.83 ± 0.89 servings respectively, *p* = 0.562), consumption of both categories was significantly higher than one USDA serving.

### Snack intake by child weight status

Independent samples test results showed that the only food group for which intake differed significantly between the overweight/obese and underweight/healthy weight categories was milk. The overweight/obese children consumed more milk than the underweight/healthy weight children (0.86 ± 0.48 vs. 0.72 ± 0.27, *p* = 0.021). The milk and fresh fruits intake of overweight/obese children was not significantly different from one USDA serving in single sample *t*-test analysis, while the underweight/healthy weight group’s intake was significantly lower than one USDA serving. Table 3 presents the data for snack intake by child weight status.

**Table 3:** Intake (mean ± SD) by weight category per snacking episode in number of servings, and comparison to one USDA serving

Food group	Under/healthy weight			Overweight/obese			*p*-value^b^
	n^a^	Mean intake ± SD	*p*-value^c^	n	Mean intake ± SD	*p*-value^c^	
Milk	40	0.72 ± 0.27	<0.000**	15	0.86 ± 0.48	0.288	0.021*
Fruits & Vegetables Overall	39	0.77 ± 0.33	<0.000**	14	0.76 ± 0.37	0.032*	0.644
Fresh fruits	32	0.64 ± 0.35	<0.000**	8	0.78 ± 0.32	0.092	0.860
Canned fruits	30	0.90 ± 0.54	0.327	13	0.80 ± 0.52	0.206	0.761
Grains & Breads Overall	37	2.06 ± 0.95	<0.000**	14	1.97 ± 0.75	<0.000**	0.658
Low sugar/low fat^d^	30	1.68 ± 0.70	<0.000**	14	1.91 ± 0.74	0.001**	0.649
High sugar/high fat^e^	33	2.14 ± 1.17	<0.000**	9	2.01 ± 0.82	0.006**	0.296
Meat and Meat Alternates^f^	24	2.29 ± 2.14	0.007**	10	1.97 ± 1.13	0.025*	0.100
Other Foods^g^	19	1.27 ± 1.06	0.277	8	1.81 ± 1.10	0.075	0.954

Statistics are based on all observations with valid data

^a^n represents the total number of children analyzed per food group. Milk was the only food group offered daily, for which all participating children had a record of intake

^b^ *p*-value is referent to differences in mean intake between the under/healthy weight group and the overweight/obese group

^c^ *p*-value is referent to differences in mean intake from one USDA serving

^d^Low sugar/low fat grains and breads included breakfast cereal, wheat crackers, wheat bagels, whole wheat breads

^e^High sugar/high fat grains and breads included cookies, graham and animal crackers, cupcakes and cakes

^f^Meat and meat alternates included cheeses, yogurt, eggs, deli meats, and peanut butter

^g^Other foods included cream cheese, cheese dip, and ice cream

## Discussion

The first aim of this study was to examine the snack intake of minority preschool children enrolled in the Head Start program in relationship to USDA serving size guidelines for reimbursable snacks. The minority preschool children in our population consumed significantly less than one USDA snack serving of milk and fruits and vegetables, while eating significantly more than one serving of grains and breads, meat and meat alternates, and other foods. We also observed a higher intake of canned fruits over fresh fruits.

The literature suggests that the intake pattern of our participants, if consistent throughout other meals during the day, could be obesity promoting. An inverse association exists between fruit and vegetable intake and body weight, with a potential effect in reducing the risk for childhood obesity [35, 36]. Adequate milk intake is also associated with a reduced risk for overweight and obesity [37, 38]. On the other hand, foods belonging to the grains and breads, meat and meat alternates, and other foods groups tend to be higher in calories, and many times in fats and added sugars, than milk and fruits and vegetables. The observed elevated intake when compared to USDA guidelines of snack foods that tend to be higher in calories is consistent with other research. For example, Ford et al. while examining changes in dietary intake among 2- to 6-year-old children from 1989 to 2008 found a significant increase in total daily energy intake by 109 kcal in parallel with an increase in foods high in added sugars, solid fats, and sodium in the preschooler diet [39]. Poti and Popkin examined trends in energy intake in children aged 2 to 18 years old by eating location and food source and found an increase in total daily energy intake by 179 kcal from 1977 to 2006, combined with an increase in foods eaten away from home, which have been associated with a higher energy density and lower nutritional quality [40]. Portion size for energy dense foods has also increased over time, and has been related to excess energy intake by children of all ages [41–43]. Therefore an elevated intake of energy dense foods, combined with larger portion sizes, may negatively affect diet quality and contribute excess calories placing children at risk for excess weight in the future.

The second aim of this study was to investigate differences in snacking by child weight status. Our sample had a higher prevalence of overweight/obesity (27.3 %) compared to the reported national level of 22.8 % for this age group [1]. Results from this study did not identify any significant relationship between the observed snack intake and child weight status. Other factors not included in this analysis such as foods from other meals, which contribute to approximately 70 % of total daily energy intake in children ages 2 to 5 years [25] may be influencing body weight in this sample, The Head Start sites included in this study are located in low-income neighborhoods, presenting high unemployment and crime rates, which may also have contributed to the increased prevalence of obesity. Lower socioeconomic status neighborhoods are at a higher risk of obesity [44, 45]. Overall our findings are consistent with previous studies, which also could not establish a relationship in preschool children between portion size, healthfulness, and obesity, although researchers report this behavior presents itself as obesity promoting [6, 46].

A significantly higher intake of milk by overweight/obese children when compared to the underweight and healthy weight group was observed. However, neither of the two groups exceeded the intake of one USDA serving for milk. Dairy products are the most important source of calcium in children’s diets and studies with children demonstrated that a higher intake of dairy products is negatively associated to body weight [37, 38, 47]. Our findings did not support the findings from other studies. While overweight/obese children’s intake of milk was not significantly different from one USDA serving, underweight/healthy weight children’s milk intake stayed significantly below it. Milk served at the Head Start centers included in our sample was 2 % reduced fat. Studies showing a protective effect of milk against overweight and obesity usually compare low fat versus whole milk. This may be a reason why our study did not find similar results. Milk remains an important food component on the diets of children and there is a need to meet recommendations for milk intake.

Findings also suggest that Head Start children are consuming significantly more than one USDA serving of grains and breads, whether offered healthy or less healthy options. Data supports the need to encourage the consumption of healthier grains and breads for snacks at preschool settings serving minority low-income children. The literature promotes offering only healthier options of such food items in preschool menus. [48]. Food preferences develop mostly during early childhood [39], making this group a potential target for nutrition interventions aimed at promoting healthy eating habits. Skinner et al. demonstrated that newly tasted foods are significantly more likely to be accepted between children 2 to 4 years old than by children 8 years old [49]. Other studies confirm the same finding: the earlier a food is introduced, the more likely it is that the child will like and consume such food item [50–52]. The observed higher intake of less healthy snacks when such were offered instead of healthier snack options, independently of weight category, suggests that there is a need to encourage preschool centers to offer a variety of healthy food items for snacks, including fresh fruits and vegetables and low fat or fat-free milk.

The convenience small sample size for this study should not be overlooked as a limitation, however little is known about the snacking habits of minority preschool children when eating at the preschool sites. Moreover, the homogeneity of the sample concerning socioeconomic status, ethnicity, education level, and neighborhood surroundings reduce the generalizability of the results yet provide insight to a vulnerable population. As a strength, in the present study snack intake was recorded through direct observation by trained dietetic interns, reducing the likelihood of over or under reporting common when dietary recalls are used. It should be pointed out that this study only considered one eating episode throughout the entire day. Other meals served at the Head Start centers were not observed, and no information was collected about the snack quality and quantity when the preschoolers were at home. This deserves further investigation.

## Conclusions

Findings from this study suggest that low-income minority preschool children are consuming larger serving sizes when offered less healthy versus healthier snack food items while eating at preschool. This snacking pattern did not show any significant relationship to the current weight status of the children. Future studies with larger sample sizes examining snacking differences in overweight/obese and underweight/healthy weight preschool children are encouraged. Studies considering more eating episodes throughout the day to give a better understanding of the children’s intake are needed. Efforts to provide healthful snack foods at the preschool setting will promote continued exposure to healthy foods. Over time this may lead to an increased acceptance of such food items and prevention of obesity.

## Abbreviations

BMI: Body mass index
CDC: Centers for Disease Control and Prevention
DI: Dietetic interns
NHANES: National Health and Nutrition Examination Survey
USDA: United States Department of Agriculture
